# Shifting national surveillance of Shigella infections toward geno‐serotyping by the development of a tailored Luminex assay and NGS workflow

**DOI:** 10.1002/mbo3.807

**Published:** 2019-03-28

**Authors:** Eleonora Ventola, Bert Bogaerts, Sigrid C. J. De Keersmaecker, Kevin Vanneste, Nancy H. C. Roosens, Wesley Mattheus, Pieter‐Jan Ceyssens

**Affiliations:** ^1^ National Reference Centre of Salmonella and Shigella Brussels Belgium; ^2^ Department of Biology and Biotechnology “C. Darwin” “Sapienza” Università di Roma Rome Italy; ^3^ Transversal activities in Applied Genomics Brussels Belgium

**Keywords:** Luminex, multiplex, public health surveillance, sequencing, Shigella

## Abstract

The phylogenetically closely related *Shigella* species and enteroinvasive *Escherichia coli* (EIEC) are responsible for millions of episodes of bacterial dysenteriae worldwide. Given its distinct epidemiology and public health relevance, only *Shigellae* are subject to mandatory reporting and follow‐up by public health authorities. However, many clinical laboratories struggle to differentiate non‐EIEC, EIEC, and *Shigella* in their current workflows, leading to inaccuracies in surveillance and rising numbers of misidentified *E. coli* samples at the National Reference Centre (NRC). In this paper, we describe two novel tools to enhance *Shigella* surveillance. First, we developed a low‐cost Luminex‐based multiplex assay combining five genetic markers for species identification with 11 markers for serotype prediction for *S. sonnei* and *S. flexneri* isolates. Using a test panel of 254 clinical samples, this assay has a sensitivity of 100% in differentiation of EIEC/*Shigella* pathotype from non‐EIEC strains, and 68.7% success rate in distinction of Shigella and EIEC. A novel, and particularly successful marker was a *Shigella*‐specific deletion in the spermidine acetyltransferase gene *speG*, reflecting its metabolic decay. For *Shigella* serotype prediction, the multiplex assay scored a sensitivity and specificity of 96.6% and 98.4%, respectively. All discrepancies were analyzed with whole‐genome sequencing and shown to be related to causative mutations (stop codons, indels, and promoter mutations) in glycosyltransferase genes. This observation spurred the development of an in silico workflow which extracts the *Shigella* serotype from Next‐Generation Sequencing (NGS) data, taking into account gene functionality. Both tools will be implemented in the workflow of the NRC, and will play a major role in the shift from phenotypic to genotyping‐based surveillance of shigellosis in Belgium.

## 1 INTRODUCTION

*Shigellae* are facultative intracellular pathogens and the etiological agents of bacillary dysentery or shigellosis (Croxen et al., 2013; Gomes et al., 2016). Shigellosis affects annually 164.7 million people, and results in a high mortality among children aged 1–4 years in low‐ and middle‐income countries (Kotloff, Riddle, Platts‐Mills, Pavlinac, & Zaidi, 2017). In western countries, *Shigella* infections were traditionally mostly travel‐related, but recent surveillance data from the United Kingdom indicate a shift to domestically circulating strains (Aragón et al., 2007; Baker et al., 2015), some of which are increasingly resistant to ciprofloxacin and azithromycin.

The *Shigella* genus is subdivided into four species based on their antigenic properties: *S. sonnei*,* S. boydii*,* S. dysenteriae*, and *S. flexneri*, each having different subtypes based on variations in the O‐antigen of the LPS layer (Edwards & Ewing, 1986). This classification does not reflect its evolutionary history as phylogenetic analyses clearly cluster *Shigella* species within the *Escherichia coli* species (Chen et al., 2014; Edwards, Logan, Langham, Swift, & Gharbia, 2012; Escobar‐Páramo, Giudicelli, Parsot, & Denamur, 2003; Pettengill, Pettengill, & Binet, 2016). In particular, enteroinvasive *E. coli* (EIEC) lineages have been identified as the direct evolutionary ancestor of *Shigella*, by having acquired a large F‐type plasmid (pINV) that encodes the molecular machinery required for invasion, survival, and diffusion of the bacterium within the host (Sansonetti, Kopecko, & Formal, 1982; Yang et al., 2005). Phylogenetic studies suggest this acquisition occurred multiple times in independent events (Hazen et al., 2016; Pettengill et al., 2016), upon which *Shigella* spp. evolved to a strictly human pathogen because of intense gene decay. This is reflected by decreased metabolic activity, increased disease severity, and decreased infectious dose (DuPont et al., 1971; Prosseda et al., 2012). Specific surveillance and differentiation of *Shigella* spp. from non‐EIEC remains therefore warranted from a medical and public health perspective.

**Figure 1:**
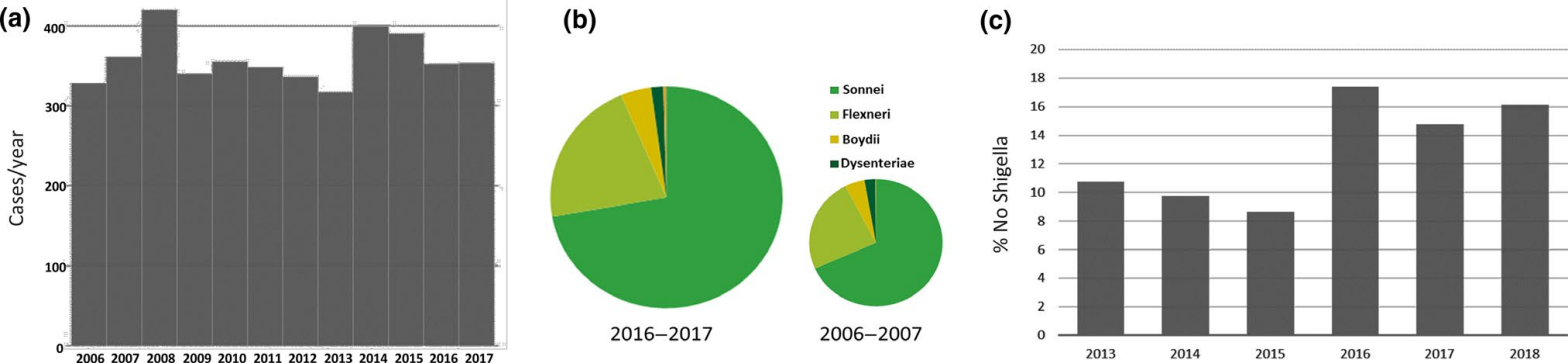
Key statistics of the Belgian National Reference Centre for Shigellosis. (a) Evolution of submitted samples in absolute numbers for the period 2006–2017. (b) *Shigella* species distribution in 2016–2017, as compared to 2006–2007. (c) Annual percentage of submitted samples that were confirmed as not being *Shigella* spp. by lack of agglutination and biochemical testing for the period 2013–2018 (data until September 2018)

National surveillance in Belgium is performed by the National Reference Centre for Shigellosis (NRCS), which receives annually approximately 400 *Shigella* cultures on a voluntary basis from peripheral laboratories (Figure 1a). Of the 2,066 confirmed *Shigella* strains received in the period 2013–2018, 72.1% were *S. sonnei*, 21.9% *S. flexneri*, 4.3% *S. boydii*, and 1.7% *S. dysenteriae* with a serotype distribution that has been stable for more than a decade (Figure 1b). Notably, the number of false‐positive *Shigella* cultures has increased substantially since 2015 as clinical laboratories increasingly rely on MALDI‐TOF for bacterial identification, which fails to properly differentiate *Shigella* from *E. coli* (Figure 1c, Khot & Fisher, 2013). *Shigella* spp. are traditionally typed using biochemical, mobility and serological assays, which are time consuming and error prone through possible cross‐reactions of O‐antigens between *E. coli* and *Shigella* (Liu et al., 2008; Sun et al., 2011). Molecular PCR methods have been described for identification and geno‐serotyping of *Shigella* spp. (Dutta et al., 2001; Gentle, Ashton, Dallman, & Jenkins, 2016; Li, Cao, et al., 2009; Sun et al., 2011), but either have limited resolution or are not cost‐effective to be implemented in routine surveillance. Some western countries have introduced whole‐genome sequencing (WGS) for *Shigella* surveillance, delivering SNP‐level discriminatory power (Chattaway et al., 2017; Dallman et al., 2016; McDonnell et al., 2013). However, wide implementation of Next‐Generation Sequencing (NGS) in national surveillance programs is hampered by budgetary limitation, a lack of bioinformatics expertise, and the extensive validation which is required at NRCs which are working under a quality system (Rossen, Friedrich, & Moran‐Gilad, 2018).

Here, we present a novel two‐step surveillance approach for *Shigella* surveillance. First, we developed a low‐cost Luminex‐based multiplex that combines species identification and subtyping of *S. flexneri* in a single test, allowing feedback to the clinical lab within 48 hr for 95% of submitted samples. This method is based on a modular multiplex oligonucleotide ligation‐PCR procedure (MOL‐PCR), using commercially available MagPlex™‐TAG microspheres for detection (Appendix 1; Ceyssens et al., 2016; Wuyts, Roosens, Bertrand, Marchal, & De Keersmaecker, 2015). Additionally, we present a workflow for extraction of *Shigella* spp. serotypes based on NGS data. We validated both arms by retrospectively analyzing 254 serotyped isolates, 16 confirmed EIEC strains, and publicly available sequence data.

## 2 METHODS

### 2.1 Bacterial strains, traditional typing, and genomic DNA extraction

In Belgium, peripheral clinical laboratories collect *Shigella* isolates from human patients and send them voluntarily to the NRCS for identification using Triple Sugar Iron Agar (TSI, Biotrading, NL) and serotyping by slide agglutination using commercially available monovalent antisera (Denka Seiken CO, UK; Appendix 2). Confirmed EIEC isolates were acquired from the “Centre for Infectious Disease Control” (RIVM, The Netherlands). Bacterial cultures were grown overnight at 37°C on Mueller–Hinton agar (Bio‐Rad). For DNA extraction, either a single colony was added to 200 μl of InstaGene™ Matrix (Bio‐Rad) and placed in a thermal cycler (56°C for 25 min, 99°C for 8 min, cooled to 4°C). The mixture was spun (14,000 g, 1 min) and the supernatant was used immediately or stored at −20°C. Alternatively, gDNA was extracted semi‐automatically using the MgC Bacterial DNA Kit™ with 60 μl elution volume (Atrida, NL), according to the manufacturer's instructions for gram‐negative bacteria.

### 2.2 MOL‐PCR using Luminex xTAG beads

For all targeted genes, upstream and downstream probes were designed targeting 35–45 bp conserved regions with maximal conservation and accessibility using OligoAnalyzer 3.1 (Table 1). Upstream probes are equipped with an internal anti‐TAG sequence compatible with the anti‐TAG of the MagPlex™ beads, while universal T7 and T3 primer sequences were added to the 5′ and 3′ ends of upstream and downstream probes, respectively. Downstream probes were 5′‐phosphorylated.

**Table 1:** Luminex probes designed using published targets

Purpose	Targeted gene	Probe	Sequence	MTAG	Reference
DNA extraction control	16S rRNA	Up	*TAATACGACTCACTATAGGG* *GTAAGAGTATTGAAATTAGTAAGA*TCCGGCCGGGAACTCAAAG	A066	Ceyssens et al. (2016)
		Down	GAGACTGCCAGTGATAAAC*TCCCTTTAGTGAGGGTTAAT*		
Identification of *Shigella* spp. and differentiation from *Escherichia coli*	*ipaH*	Up	*TAATACGACTCACTATAGGG* TTTGTTAGAATGAGAAGATTTATGTCCATCAGGCATCWGAAGGC	A075	Venkatesan et al. (1991)
		Down	CTTTTCGATAATGATACCGGC*TCCCTTTAGTGAGGGTTAAT*		
	*invC*	Up	*TAATACGACTCACTATAGGG* *AGTAGAAAGTTGAAATTGATTATG*CTGCCCAGTTTCTTCATACG	A012	Ojha et al. (2013)
		Down	CAAGTCGGCCGTGGATTATT*TCCCTTTAGTGAGGGTTAAT*		
	*speG*	Up	*TAATACGACTCACTATAGGG* AATGAAATAGTGTTAAATGAGTGTATGCCAAGCGCCCACAGTG	A074	Barbagallo et al. (2011)
		Down	TTAAGCTACGCCCGCTGGA*TCCCTTTAGTGAGGGTTAAT*		
	*cadA*	Up	*TAATACGACTCACTATAGGG* AGTAAGTGTTAGATAGTATTGAATCATGGCAACGACAAATTAAAGG	A038	Prosseda et al. (2007)
		Down	CGAAGTAGAAACCATTGCGC*TCCCTTTAGTGAGGGTTAAT*		
	*lacY*	Up	*TAATACGACTCACTATAGGG* AATGTAAAGTAAAGAAAGTGATGAGTATGTTATTGGCGTTTCCTG	A044	Løbersli, Wester, Kristiansen, and Brandal (2016)
		Down	CACCTACGATGTTTTTGA*TCCCTTTAGTGAGGGTTAAT*		
*S. sonnei geno‐serotyping*	*wbgZ*	Up	*TAATACGACTCACTATAGGG* ATTGTGAAAGAAAGAGAAGAAATTGTAATGTACTCGGTTCTTCGG	A014	Ojha et al. (2013)
		Down	GCTCTGTCGTGCCGTTGTTTG*TCCCTTTAGTGAGGGTTAAT*		
*S. flexneri geno‐serotyping*	*rfc*	Up	*TAATACGACTCACTATAGGG* *AGTGAATGTAAGATTATGTATTTG* *CTTTACATGGTCGGATCAC*	A013	Ojha et al. (2013)
		Down	GCAGTGAAGATTCTGACTCT*TCCCTTTAGTGAGGGTTAAT*		
	*wzx1‐5*	Up	*TAATACGACTCACTATAGGG* TTTGTGTGTTATTGTAATTGAGATTTCGGCGAAAAGTGGAACAG	A067	Gentle et al. (2016)
		Down	CATTATTCCGGTGCTGCAAT*TCCCTTTAGTGAGGGTTAAT*		
	*wzx6*	Up	*TAATACGACTCACTATAGGG* TTTGTTGTTAAGTATGTGATTTAGGCGATCATTTCAACTTCAAC	A063	Gentle et al. (2016)
		Down	GGTAATTCTAACTATATTGGGC*TCCCTTTAGTGAGGGTTAAT*		
	*gtrI*	Up	*TAATACGACTCACTATAGGG* TTGTGTAGTTAAGAGTTGTTTAATTGCTAACAGCCCAATTGTATG	A036	Gentle et al. (2016)
		Down	GAGGCATATTTTAGAGAATGG*TCCCTTTAGTGAGGGTTAAT*		
	*gtrII*	Up	*TAATACGACTCACTATAGGG* TTTAAGTGAGTTATAGAAGTAGTAGACTCAGGAAATATGCTCTC	A029	Gentle et al. (2016)
		Down	CATGAGCGCAGACACTTTTGG*TCCCTTTAGTGAGGGTTAAT*		
	*gtrIV*	Up	*TAATACGACTCACTATAGGG* TGAGTAAGTTTGTATGTTTAAGTAGGCCATAACACCTTTCATGAATG	A065	Gentle et al. (2016)
		Down	GGATCAGACAGTTCTCACATG*TCCCTTTAGTGAGGGTTAAT*		
	*gtrV*	Up	*TAATACGACTCACTATAGGG* AGAGTATTAGTAGTTATTGTAAGTTAACTTGCTCTTTCCACC	A057	Gentle et al. (2016)
		Down	CGTAATCTGGGAGTGGGGTAA*TCCCTTTAGTGAGGGTTAAT*		
	*gtrX*	Up	*TAATACGACTCACTATAGGG* AATTAGAAGTAAGTAGAGTTTAAGGTCCAAGCCAATATAACAAATG	A056	Gentle et al. (2016)
		Down	CTCACTGGTATTTATCATTG*TCCCTTTAGTGAGGGTTAAT*		
	*gtr1c*	Up	*TAATACGACTCACTATAGGG* AATTGAGAAAGAGATAAATGATAGGTCATACGCTTTCTCACGAAC	A072	Gentle et al. (2016)
		Down	CTTAGGTTCAAATGGGTTAC*TCCCTTTAGTGAGGGTTAAT*		
	*oac*	Up	*TAATACGACTCACTATAGGG* ATTAAGTAAGAATTGAGAGTTTGAAACTGCTTTGACACGGCAAGG	A021	Gentle et al. (2016)
		Down	CTTGTGGCAGCTATGATGGTT*TCCCTTTAGTGAGGGTTAAT*		

*Note*.

The universal T7 and T3 primer sequences are indicated in italics. Anti‐TAG sequences compatible with the indicated MagPlex‐TAG Microspheres (MTAG) are underlined.

Our MOL‐PCR approach has been described in detail elsewhere (Wuyts et al., 2015). Briefly, all reactions were assembled in cooled 96‐well plates in a 10 μl reaction volume containing 2 nM of each probe, 2 U of Taq DNA Ligase (New England Biolabs, Ipswich, MA), 1× Taq DNA ligase buffer, 2 μl of DNA template, and nuclease‐free water. Ligation was performed by initial denaturation (95°C, 10 min), followed by 25 cycles of ligation (58°C, 30 s) and denaturation (96°C, 25 s). Three microliters of the ligation product was amplified in a 10 μl PCR containing 0.25 U of HotStartTaq DNA polymerase (Qiagen, Hilden, Germany), 1× DNA polymerase buffer, 125 nM T7 primer, 500 nM 5′‐biotin‐T3 primer, and 200 μM dNTPs. Reaction conditions were 15 min of denaturation at 95°C, followed by 35 cycles of 94°C (30 s), 60°C (30 s), and 72°C (30 s), and a final extension step at 72°C for 5 min.

Hybridization of the PCR product to colored microspheres was performed in a volume of 20 μl per reaction, with MagPlex™‐TAG microspheres (750 beads/target) added to 0.1 M Tris–HCl, pH 8.0/0.2 M NaCl/0.08% Triton‐X. To this mixture, 5 μl of PCR product was added, followed by a denaturation step (90 s at 96°C) and 30 min of hybridization at 37°C. Subsequently, 100 μl of a reporter mix containing 4 μg/ml streptavidin‐R‐phycoerythrin (Life Technologies) was added, and the samples were incubated for 15 min at 37°C in the dark. Subsequent read‐out was performed at 37°C using 100 μl of these samples, on a MAGPIX device with a minimal bead count of 50 microspheres/target (Wuyts et al., 2015). For each marker, the signal‐to‐noise (S/N) ratios were calculated by dividing the median fluorescence intensity (MFI) by the corresponding MFI of the NC. During assay design, an S/N ratio ≥2.0 indicated positive identification.

### 2.3 Whole genome sequencing and in silico serotyping

Genomic DNA was prepared using MgC Bacterial DNA Kit™ with 60 μl elution volume (Atrida, NL), following the manufacturer's instructions. Sequencing libraries were constructed using the Illumina Nextera XT DNA sample preparation kit and subsequently sequenced on an Illumina MiSeq instrument with a 250‐bp paired‐end protocol (MiSeq v3 chemistry) according to the manufacturer's instructions.

Sequence variants were collected for *wzx1‐5*,* wzx6*,* gtrI*,* gtrII*,* gtrIV*,* gtrV*,* gtrX*,* gtr1c*,* oac*, and *opt*, and also for the *ipaH* and *rfc* gene sequences. Raw reads were trimmed using Trimmomatic v0.36 (Bolger, Lohse, & Usadel, 2014) with the following settings: “ILLUMINACLIP: NexteraPE‐PE.fa:2:30:10,” “LEADING:10,” “TRAILING:10,” “SLIDINGWINDOW:4:20” and “MINLEN:40.” Afterward SRST2 v0.2.0 using default settings was employed to detect the presence of genes using trimmed reads as input against the constructed sequence database (Inouye et al., 2014). A variant calling‐based approach was then used to specifically detect stop and frameshift mutations leading to inactivation in the detected genes as follows. Trimmed reads were mapped against the sequence of every identified gene using bowtie2 v2.3.0 with the “–very‐sensitive‐local” option enabled (Langmead & Salzberg, 2012). The resulting SAM file was then converted into an indexed BAM file using SAMtools view v1.3.1, followed by SAMtools sort and SAMtools index (Li, Handsaker, et al., 2009). Afterward, a pileup was generated using SAMtools mpileup with output format set to “VCF,” followed by variant calling by BCFtools call v1.6 with the following options: “–consensus‐caller,” “–variants‐only,” and “–ploidy 1” (Li, 2011). Variants that were covered by <10 reads or variants that were not covered by at least one forward and one reverse read were removed using BCFtools filter (Danecek & McCarthy, 2017). Indels were normalized and duplicates removed using BCFtools norm with the option “–rm‐dup both.” Finally, the functional effect of the mutations was determined using BCFtools csq v1.9.30 (commit: g983f7da) with the option “–local‐csq” enabled. Genes that contained a stop codon and/or a frameshift were considered to be not expressed for the determination of the serotype. Mutations in the *gtr* promotor were detected similarly by first mapping trimmed reads against a 381 bases‐long region covering the *gtr* promotor and initial coding sequence (accession number KT988057.1). Read mapping and variant calling were done as described before but variant filtering was slightly more strict: minimal depth 10×, minimal forward depth 1×, minimal reverse depth 1×, minimal SNP quality 25, minimal mapping quality 30, minimal *Z*‐score of 1.96, and minimal Y‐multiplier of 10 as described elsewhere (Kaas, Leekitcharoenphon, Aarestrup, & Lund, 2014). The promotor was considered to be wild type if there were no filtered mutations inside the ‐35 box or the ‐10 TA box. Otherwise the *gtrX* promoter was considered as not wild type and the *gtrX* gene as not expressed for the determination of the serotype. The profiles described in Sun et al. (2011) were then used as a decision system to classify the serotype.

## 3 RESULTS

### 3.1 Multiplex target selection and design

**Figure 2:**
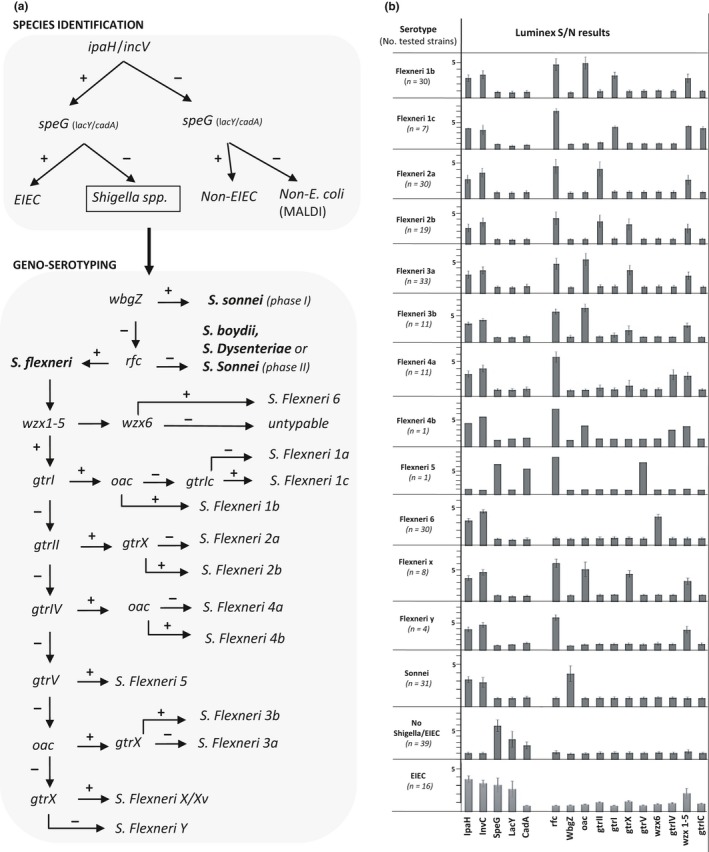
In‐house molecular (MOL)‐PCR‐based Luminex assay for *Shigella* typing. (a) Decision tree for the developed MOL‐PCR assay for detection and subtyping of *Shigella* spp. (b) Graphical representation of raw Luminex data for tested species and serotypes during test validation. The read‐out is scored as the median fluorescence intensity, which is converted to signal‐to‐noise ratios (S/N) for allele calling. The single available isolate of *S. flexneri* 5 was confirmed as *Escherichia coli* based on whole‐genome sequencing

To introduce molecular testing in national *Shigella* surveillance, we designed a specific multiplex assay for identification, differentiation, and subtyping of *Shigella* spp. from cultured strains. Our strategy was based on converting known molecular markers into a MOL‐PCR assay with read‐out on a Luminex MAGPIX^®^ platform, allowing multiplex detection of up to 50 genes in a single well (Table 1). For identification of the EIEC/*Shigella* pathotype, we targeted the invasive plasmid antigen H (*ipaH*) and the plasmid invC (Ojha, Yean, Ismail, & Singh, 2013; Venkatesan, Buysse, & Hartman, 1991). To distinguish EIEC from *Shigella*, we inferred the presence of *lacY* (Pavlovic et al., 2011), *cadA* (Prosseda et al., 2007) and a Shigella‐specific deletion 19_20delGT in *speG* (Prosseda G, personal communication). Next, we included probes targeting *wbgZ* and *rfc* for identification of *S. sonnei* and *S. flexneri*, respectively (Ojha et al., 2013). Finally, we adapted a previously described multiplex PCR assay for serotyping of *S. flexneri* that targets genes for O‐antigen synthesis or modification (Gentle et al., 2016; Sun et al., 2011) into to a Luminex‐compatible format (Table 1). A decision tree to interpret the results of the final assay can be found in Figure 2a. A probe targeting the *opt* gene, responsible for addition of phosphoethanolamine to L‐rhamonse II or III, leading to Flexneri variants 4av, Xv, and Yv (Sun et al., 2012), was not included as no positive control samples were present in our collection. Genetic serotyping of *S. boydii* and *S. dysenteriae* was omitted from the current assay as this would have required the inclusion of 31 additional targets, substantially increasing the reaction cost to cover only a minority of samples submitted in Belgium (<5%, Figure 1b).

### 3.2 Luminex‐based species identification

To validate the assay and assess its performance in distinguishing *Shigella* from either non‐EIEC and EIEC, we retrospectively analyzed 215 samples sent to the Belgian NRC between 2013 and 2018 that had been routinely typed using traditional biochemical and serological methods (Appendix 2). We randomly selected isolates of Sonnei (*n *=* *31, of which 26 Phase I Sonnei), Flexneri 1b (*n *=* *30), 2a (*n *=* *30), 2b (*n *=* *19), 3a (*n *=* *33), 3b (*n *=* *11), 4a (*n *=* *11), and 6 (*n *=* *30). Serotypes 1c (*n *=* *7), 4b (*n* = 1), 5a/b (*n* = 1), X (*n* = 8), and Y (*n* = 4) are underrepresented in the NRCS collection in comparison to other serotypes, and all available isolates were included in this study. To this collection, we added 33 isolates which had a negative identification for *Shigella* spp., 16 confirmed EIEC strains, and six untypable isolates exhibiting nonspecific agglutination reactions.

During the test phase of the assay, we detected false‐positive signals in 5.1% (13/254) of the tested isolates, due to an elevated background (Appendix 2), which disappeared upon re‐extraction of their gDNA (data not shown). In 12 of 13 cases, elevated backgrounds were observed in samples extracted by the MgC Bacterial DNA Kit™, suggesting a better compatibility of the InstaGene^®^ Matrix extraction method with Luminex‐based read‐out. Secondly, in 14.1% (36/254) of the tested samples, an elevated background signal in the No Template Control (NTC) sample lead to false‐negative results (Appendix 2). This elevated signal disappeared upon replacing the NTC with 10 pg of *S. enterica* DNA (data not shown). As an additional measure to increase the test robustness, S/N values with of at least twice the baseline value of other probes in the same sample were considered positive throughout the study.

After these optimizations, all samples confirmed as *S. sonnei* or *S. flexneri* by traditional methods (215/215) were positive for either the *ipaH* (99.5%) or the *inv*C (95.2%) probe, and negative for *speG*,* lac*Y, and *cad*A. Similarly, all 39 isolates not identified as *Shigella* (i.e., including untypable samples) were negative for *ipa*H and *inv*C. These strains were positive for either *speG* (37/39), *lac*Y (19/39), and/or *cad*A (21/39) (Figure 2). One isolate negative for the *speG* probe (S13BD01340) was identified as *Citrobacter freundii* by MALDI‐TOF, leading to a sensitivity of 97.4% for this probe in identifying *E. coli* in our test set. The other *speG* negative isolate (S17BD01771) tested positive for *cadA*, leading to 100% sensitivity in detecting *E. coli* with all probes combined. Not unexpectedly, the 16 examined EIEC strains gave an intermediate profile in the multiplex (Appendix 2, Figure 2). Two EIEC reference strains and 13 of 14 clinical EIEC isolates were positive for *ipa*H and *inv*C, while the presence of *speG*,* lac*Y, and *cad*A was detected in 11/16, 6/16, and 0/16 of strains, respectively.

### 3.3 Luminex‐based serotyping and discrepance analysis

The algorithm for deriving *Shigella* serotypes from the multiplex data is shown in Figure 2a. The multiplex assay determined correctly the serotype of 26/26 *S. sonnei* Phase I and 176/185 (95.1%) *S. flexneri* samples, with 100% concordance between genotyping and classical typing for Flexneri Types 1b, 1c, 2a, 2b, 3a, 6, and Y (Appendix 2). As expected, isolates belonging to Sonnei Phase II (5/5) could not be detected. We employed NGS to evaluate the 10 discordant Flexneri isolates in more detail (Table 2), which allowed to characterize the genes responsible for O‐antigen synthesis or modification at a much higher resolution. We identified explanatory indels and frameshift mutations in *oac*,* gtr*I, and *gtr*IV in six strains, impeding their function (Gentle et al., 2016). Moreover, we detected promoter mutations upstream of the *gtr* operon in four strains, suggesting decreased expression levels resulting in the *S. flexneri* 3b serotype. A peculiar result was observed for strain S16BD02240, which was previously typed as the only *S. flexneri* 5 isolate in Belgium. While the species identification panel detected *speG* and not *ipaH* or *inv*C, the serotype probes rfc and gtrV were positive (Appendix 2). Closer inspection of sequencing results revealed the insertion of a phage‐encoded gtrV protein in an *E. coli* background, leading to the *E. coli* O13/O135:H11 serotype (Knirel et al., 2016).

**Table 2:** NGS analysis of *Shigella flexneri* strains with discrepant results between serotyping and Luminex‐based typing

Strain ID	Serotype		Remarks
	Phenotype	Luminex	
S15BD09453	flexneri 3b	flexneri 1b	Indel detected in *gtr*I at position 340
S13BD04017	flexneri 3b	flexneri 3a	Gtr operon promoter mutations at positions ‐6, ‐7, ‐12, ‐13, ‐14, ‐17, ‐18, and ‐19
S14BD01714	flexneri 3b	flexneri 3a	Gtr operon promoter mutations at positions ‐6, ‐7, ‐12, ‐13, ‐14, ‐17, ‐18, and ‐19
S15BD06353	flexneri 3b	flexneri 3a	Gtr operon promoter mutations at positions ‐6, ‐7, ‐12, ‐13, ‐14, ‐17, ‐18, and ‐19
S15BD08204	flexneri 3b	flexneri 3a	Gtr operon promoter mutations at positions ‐6, ‐7, ‐12, ‐13, ‐14, ‐17, ‐18, and ‐19
S16BD02240	flexneri 5	flexneri 5a/coli	gtrV/rfc detected, *ipa*H absent
S14BD02502	flexneri x	flexneri 3a	Indel detected in *oac* at position 543
S17BD07654	flexneri x	flexneri 3a	Indel detected in *oac* at position 718
S14BD01142	flexneri x	flexneri 3a	Frameshift detected in *oac* at position 346
S14BD01131	flexneri x	flexneri 3a	Frameshift detected in *oac* at position 346

### 3.4 NGS‐based serotyping

**Figure 3:**
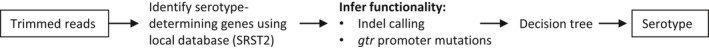
Schematic overview of NGS workflow. Technical details are described in the Methods section

To enhance future workflows, we designed a WGS‐based workflow for automated extraction of *Shigella* serotypes from NGS data that includes detection of *opt*,* wzx*,* wzy*, and other known glycosyltransferase genes, enabling the detection of all currently described variants of the O‐antigen from *S. boydii*,* S. sonnei, S. dysenteriae*, and *S. flexneri* (Li, Cao, et al., 2009). To account for observed differences between phenotypes and genotypes described previously, we included the detection of TAG stop codon and frameshifts in all analyzed genes, and promotor mutations in the *gtr* operon (Figure 3). The algorithm was tested on publicly available NGS data from 135 globally collected *S. flexneri* strains (Connor et al., 2015), leading to identical serotype predictions in 127 of 135 (94.1%) of tested strains (Appendix 3). Interestingly, frameshifts (17%) and amber mutations (2.9%) were regularly detected among the 127 correctly predicted serotypes, thus showing frequent inactivation of glycosyltransferase genes. Next, we analyzed the eight deviating results using the CLC Bio Genome Workbench. In two samples, we observed low (<5×) coverage of the *opt* gene, hinting at plasmid loss in a subpopulation of the culture. Given the minimal coverage set at 10×, these genes were below our detection limit. In 3 of 8 cases, our NGS workflow failed to call *gtr* operon promoter mutations (*n *=* *1), and indels in *gtr*X (*n *=* *1) and *oac* (*n *=* *1). In the three remaining cases, no obvious explanation of the discrepancy could be detected.

## 4 DISCUSSION AND CONCLUSION

Many clinical laboratories struggle to differentiate non‐EIEC, EIEC, and *Shigella* spp. in their current workflows, although their discrimination is important for public health surveillance as only *Shigella* is subject to mandatory reporting (van den Beld et al., 2018). In order to address rising numbers of misidentified *E. coli* samples at the NRCs and to speed up the *Shigella* subtyping process, we developed a Luminex‐based multiplex assay combining species identification and serotype prediction for *S. sonnei* and *S. flexneri* isolates.

While successful positive identification of *Shigella*/EIEC is based on the well‐known target of *ipaH* (99.5% among tested strains), we describe in this study a particularly successful SNP for which a high negative predictive value (99.6%) and sensitivity (97.4%) were observed for non‐EIEC *E. coli*. This SNP causes a frameshift mutation in *speG*, encoding the enzyme spermidine acetyltransferase responsible for the conversion of spermidine into N‐acetylspermidine. It has been demonstrated that a higher level of spermidine increases *Shigella* survival within macrophages and confers higher resistance to oxidative stress (Barbagallo et al., 2011), indicating that the loss of *speG* function is an emerging trait. As predicted, EIEC have an intermediate position as active N‐acetylspermidine is still present in most EIEC strains (68.7% in our dataset), yet intracellular spermidine tends to be higher as compared to commensal *E. coli* (Campilongo et al., 2014). Interestingly, among all non‐*Shigella* strains that were sent to the NRCS by peripheral Belgian laboratories, not a single strain with defective *speG* was detected (Appendix 2), strongly suggesting that the large majority are non‐EIEC strains.

A weakness of the current assay is the low positive predictive value for EIEC strains. A first option to cope with this is to expand the biochemical typing of ipaH positive strains, as described by van den Beld et al. (2018). Alternatively, the discriminatory power of the molecular assay can be increased by incorporating additional markers published by Australian researchers during the review process of this article (Dhakal, Wang, Lan, Howard, & Sintchenko, 2018). Their large‐scale genome comparisons identified six genetic loci separating Shigella from EIEC, which combined presence/absence led to 95.1% sensitivity. Due to the flexibility of the Luminex‐based MOL‐PCR methodology, the expansion of our assay from a 17‐ to a 23‐plex is expected to go swiftly with minimal impact on cost and handling time.

In addition to species identification, the presented Luminex assay simultaneously detects *S. sonnei* Phase I and *S. flexneri* serotypes. Two published reports on molecular geno‐serotyping report 92.6% and 97.8% concordant results between phenotypic serotyping and PCR (Gentle et al., 2016; Sun et al., 2011), comparable with the 95.1% observed in our MOL‐PCR based assay. As reported also in these studies, we also observed a robust correlation between the phenotypes and genotypes for *S. sonnei* and *S. flexneri* serotypes 1b, 1c, 2a, 2b, 3a, F6, and Y. Discrepancies are commonly caused by amber mutations, insertions, and deletions in O‐antigen synthesis or modification genes, rendering these phenotypically inactive. In our test set, these accounted for 5.4% of deviating results among tested *S. flexneri*. In the global collection of Flexneri strains analyzed by Connor et al. (2015), 19.9% of strains contained such mutations, making a strong case for using WGS data in serotype prediction instead of PCR‐based methods that only take a part of the gene into account. As a note, *opt‐*mediated O‐antigen modification was not detectable in our assay, and should be part of future updates.

In all *Shigella* species, genes for O‐antigen synthesis and modification are typically encoded on mobile elements like prophages and plasmids, and hence are unstable phenotypic markers (Connor et al., 2015; Sun et al., 2013). Recent genomic studies showed evidence of high levels of recombination among genes responsible for serotypes, limiting their use in transmission and epidemiological studies (Connor et al., 2015; Dallman et al., 2016). Therefore, it has little doubt that epidemiological surveillance of *Shigella* infections will increasingly shift to NGS, as long as allocated budgets allow this transition. Our NGS workflow is able to accurately perform serotype predictions from sequence data, and will be incorporated in future bioinformatic pipelines to allow backwards compatibility with historical results and with traditionally typed strains. In the meantime, the presented Luminex MAGPIX^®^‐based assay can provide a cost‐effective solution for fast detection and subtyping of the most prevalent *Shigella* spp. This multiplex surpasses limitations of traditional typing, and is readily implementable in clinical and public health laboratories.

## CONFLICT OF INTEREST

All authors report no conflict of interest.

## AUTHORS CONTRIBUTION

E.V. performed wet lab experiments; B.B. and K.V. performed bioinformatics; W.M., S.D.K., and N.R. provided technical expertise; P.C. designed the experiments and wrote the paper. All authors read and approved the manuscript.

## ETHICS STATEMENT

None required.

## Data Availability

Raw sequence data were submitted to the European Nucleotide Archive (ENA; EMBL‐EBI, Cambridge, UK) as accession number PRJEB30509 and study name ena‐STUDY‐Sciensano‐21‐12‐2018‐12:27:07:718‐355. Accession numbers of publicly available NGS data used in this study, and raw Luminex data are listed in Appendix 3.

## APPENDIX 1

### 1.1

*Schematic representation of the MOL‐PCR method*. When a target allele or SNP is present in the DNA extract of a bacterial strain under study, two sequence‐specific probes will be ligated in 25 consecutive cycles of denaturation and annealing. The upstream probes carry a 24 nucleotide sequence (shown in red) which binds to the anti‐TAG covalently attached to the MagPlex‐TAG microspheres (Luminex). Using universal T7 and 5′‐biotin labeled T3 primers (shown in blue), the ligated oligonucleotides are amplified, denatured, and hybridized to the corresponding beads. After short exposure to streptavidin‐R‐phycoerythrin, the luminescent signal is read by the MAGPIX^®^ machine, and S/N ratios are calculated using negative control samples. This figure is modified from Ceyssens et al. (2016).

## APPENDIX 2

### 2.1

*Overview of selected strains and Luminex results*. Genomic DNA was isolated using either Instagene^®^ Matrix (IM) or the MgC Bacterial DNA Kit™ (MC). For extraction control, we included a probe targeting 16S rRNA; The result was expressed as positive (“pass”) if the resulting MFI surpassed 500 units. Shown in the “species identification” and “serotype prediction” tabs are signal‐to‐noise (S/N) values of the different probes, using a *S. enterica* strain as negative control. A signal is considered positive if the S/N exceeds 2.0 (cells highlighted in green), or if the S/N value of the probe has a least twice the baseline value of other probes in the same sample (cells highlighted in orange). False‐positive signals caused by elevated background signals are indicated in blue. Strains marked with an asterisk gave discrepant results between serotyping and Luminex analysis, and were analyzed using NGS.

## APPENDIX 3

### 3.1

**Table:** In silico workflow results

Sample ID	NGS algorithm									Comment
	Flexneri type^a^	ipaH present	rfc present	Detected genes	Wild type P_gtr_ b	Amber mutation	Frameshift	Result	Match	
ERR042839	X	TRUE	TRUE	gtrX, opt, wzx1_5	TRUE	—	—	Xv	FALSE	*opt* detected in low (4.7×) coverage
ERR042840	Yv	TRUE	TRUE	gtrX, opt, wzx1_5	TRUE	—	—	Xv	FALSE	gtr promoter mutations not called
ERR048322	5a	TRUE	TRUE	gtrII, wzx1_5	TRUE	—	—	2a	FALSE	No obvious cause for discrepancy detected
ERR127048	4a	TRUE	TRUE	gtrIV, opt, wzx1_5	TRUE	—	—	4av	FALSE	No obvious cause for discrepancy detected
ERR217013	Yv	TRUE	TRUE	gtrX, opt, wzx1_5	TRUE	—	—	Xv	FALSE	Indel in *gtr*X not detected
ERR832473	X	TRUE	TRUE	gtrX, oac, wzx1_5	TRUE	—	—	3a	FALSE	indel oac not detected
ERR832480	5a	TRUE	TRUE	gtrV, wzx1_5	TRUE	gtrV	—	Y	FALSE	No obvious cause for discrepancy detected
ERR832481	Y	TRUE	TRUE	opt, wzx1_5	TRUE	—	—	Yv	FALSE	*opt* detected in low (2.6×) coverage
ERR048265	5b	TRUE	TRUE	gtrV, gtrX, oac, wzx1_5	TRUE	—	oac	5b	TRUE	
ERR127042	5b	TRUE	TRUE	gtrV, gtrX, oac, wzx1_5	TRUE	—	oac	5b	TRUE	
ERR127044	5b	TRUE	TRUE	gtrV, gtrX, oac, wzx1_5	TRUE	—	oac	5b	TRUE	
ERR217033	1cv	TRUE	TRUE	gtrI, gtrIC, gtrX, opt, wzx1_5	TRUE	—	gtrX	1cv	TRUE	
ERR042796	Xv	TRUE	TRUE	gtrX, opt, wzx1_5	TRUE	—	—	Xv	TRUE	
ERR042797	X	TRUE	TRUE	gtrX, wzx1_5	TRUE	—	—	X	TRUE	
ERR042799	2a	TRUE	TRUE	gtrII, wzx1_5	TRUE	—	—	2a	TRUE	
ERR042803	2a	TRUE	TRUE	gtrII, wzx1_5	TRUE	—	—	2a	TRUE	
ERR042806	2b	TRUE	TRUE	gtrII, gtrX, wzx1_5	TRUE	—	—	2b	TRUE	
ERR042810	1b	TRUE	TRUE	gtrI, oac, wzx1_5	TRUE	—	—	1b	TRUE	
ERR042811	3a	TRUE	TRUE	gtrX, oac, wzx1_5	TRUE	—	—	3a	TRUE	
ERR042814	2a	TRUE	TRUE	gtrII, wzx1_5	TRUE	—	—	2a	TRUE	
ERR042816	Y	TRUE	TRUE	wzx1_5	TRUE	—	—	Y	TRUE	
ERR042819	3b	TRUE	TRUE	oac, wzx1_5	TRUE	—	—	3b	TRUE	
ERR042821	2a	TRUE	TRUE	gtrII, wzx1_5	TRUE	—	—	2a	TRUE	
ERR042824	2a	TRUE	TRUE	gtrII, wzx1_5	TRUE	—	—	2a	TRUE	
ERR042825	2a	TRUE	TRUE	gtrII, wzx1_5	TRUE	—	—	2a	TRUE	
ERR042831	Y	TRUE	TRUE	wzx1_5	TRUE	—	—	Y	TRUE	
ERR042835	1a	TRUE	TRUE	gtrI, wzx1_5	TRUE	—	—	1a	TRUE	
ERR042837	3a	TRUE	TRUE	gtrX, oac, wzx1_5	TRUE	—	—	3a	TRUE	
ERR042841	1a	TRUE	TRUE	gtrI, wzx1_5	TRUE	—	—	1a	TRUE	
ERR042842	2a	TRUE	TRUE	gtrII, wzx1_5	TRUE	—	—	2a	TRUE	
ERR042843	3a	TRUE	TRUE	gtrX, oac, wzx1_5	TRUE	—	—	3a	TRUE	
ERR042845	Xv	TRUE	TRUE	gtrX, opt, wzx1_5	TRUE	—	—	Xv	TRUE	
ERR042849	1c	TRUE	TRUE	gtrI, gtrIC, wzx1_5	TRUE	—	—	1c	TRUE	
ERR042851	1b	TRUE	TRUE	gtrI, oac, wzx1_5	TRUE	—	—	1b	TRUE	
ERR042852	2b	TRUE	TRUE	gtrII, gtrX, wzx1_5	TRUE	—	—	2b	TRUE	
ERR042853	1c	TRUE	TRUE	gtrI, gtrIC, wzx1_5	TRUE	—	—	1c	TRUE	
ERR042855	3a	TRUE	TRUE	gtrX, oac, wzx1_5	TRUE	—	—	3a	TRUE	
ERR042860	2a	TRUE	TRUE	gtrII, wzx1_5	TRUE	—	—	2a	TRUE	
ERR042861	Xv	TRUE	TRUE	gtrX, opt, wzx1_5	TRUE	—	—	Xv	TRUE	
ERR042863	Yv	TRUE	TRUE	opt, wzx1_5	TRUE	—	—	Yv	TRUE	
ERR047236	1b	TRUE	TRUE	gtrI, oac, wzx1_5	TRUE	—	—	1b	TRUE	
ERR047239	3a	TRUE	TRUE	gtrX, oac, wzx1_5	TRUE	—	—	3a	TRUE	
ERR047294	1b	TRUE	TRUE	gtrI, oac, wzx1_5	TRUE	—	—	1b	TRUE	
ERR047306	2a	TRUE	TRUE	gtrII, wzx1_5	TRUE	—	—	2a	TRUE	
ERR047307	NA	TRUE	TRUE	gtrII, opt, wzx1_5	TRUE	—	—	NA	TRUE	
ERR047396	3a	TRUE	TRUE	gtrX, oac, wzx1_5	TRUE	—	—	3a	TRUE	
ERR047406	2a	TRUE	TRUE	gtrII, wzx1_5	TRUE	—	—	2a	TRUE	
ERR048234	1c	TRUE	TRUE	gtrI, gtrIC, wzx1_5	TRUE	—	—	1c	TRUE	
ERR048246	2a	TRUE	TRUE	gtrII, wzx1_5	TRUE	—	—	2a	TRUE	
ERR048259	4bv	TRUE	TRUE	gtrIV, oac, opt, wzx1_5	TRUE	—	—	4bv	TRUE	
ERR048261	1b	TRUE	TRUE	gtrI, oac, wzx1_5	TRUE	—	—	1b	TRUE	
ERR048285	3a	TRUE	TRUE	gtrX, oac, wzx1_5	TRUE	—	—	3a	TRUE	
ERR048286	1b	TRUE	TRUE	gtrI, oac, wzx1_5	TRUE	—	—	1b	TRUE	
ERR048287	5a	TRUE	TRUE	gtrV, oac, wzx1_5	TRUE	—	oac	5a	TRUE	
ERR048288	Y	TRUE	TRUE	gtrX, wzx1_5	TRUE	—	gtrX	Y	TRUE	
ERR048290	1b	TRUE	TRUE	gtrI, oac, wzx1_5	TRUE	—	—	1b	TRUE	
ERR048295	2b	TRUE	TRUE	gtrII, gtrX, wzx1_5	TRUE	—	—	2b	TRUE	
ERR048296	3a	TRUE	TRUE	gtrX, oac, wzx1_5	TRUE	—	—	3a	TRUE	
ERR048300	3a	TRUE	TRUE	gtrX, oac, wzx1_5	TRUE	—	—	3a	TRUE	
ERR048302	2a	TRUE	TRUE	gtrII, wzx1_5	TRUE	—	—	2a	TRUE	
ERR048304	2a	TRUE	TRUE	gtrII, wzx1_5	TRUE	—	—	2a	TRUE	
ERR048306	2a	TRUE	TRUE	gtrII, wzx1_5	TRUE	—	—	2a	TRUE	
ERR048313	2b	TRUE	TRUE	gtrII, gtrX, wzx1_5	TRUE	—	—	2b	TRUE	
ERR048315	2a	TRUE	TRUE	gtrII, wzx1_5	TRUE	—	—	2a	TRUE	
ERR048316	1b	TRUE	TRUE	gtrI, oac, wzx1_5	TRUE	—	—	1b	TRUE	
ERR048319	1a	TRUE	TRUE	gtrI, wzx1_5	TRUE	—	—	1a	TRUE	
ERR048320	X	TRUE	TRUE	gtrX, wzx1_5	TRUE	—	—	X	TRUE	
ERR048331	Y	TRUE	TRUE	wzx1_5	TRUE	—	—	Y	TRUE	
ERR048339	2b	TRUE	TRUE	gtrII, gtrX, wzx1_5	TRUE	—	—	2b	TRUE	
ERR049152	3a	TRUE	TRUE	gtrV, gtrX, oac, wzx1_5	TRUE	gtrV	—	3a	TRUE	
ERR126958	2a	TRUE	TRUE	gtrII, wzx1_5	TRUE	—	—	2a	TRUE	
ERR127015	2a	TRUE	TRUE	gtrII, wzx1_5	TRUE	—	—	2a	TRUE	
ERR127017	2a	TRUE	TRUE	gtrII, wzx1_5	TRUE	—	—	2a	TRUE	
ERR127019	3a	TRUE	TRUE	gtrX, oac, wzx1_5	TRUE	—	—	3a	TRUE	
ERR127032	1a	TRUE	TRUE	gtrI, wzx1_5	TRUE	—	—	1a	TRUE	
ERR127034	1c	TRUE	TRUE	gtrI, gtrIC, wzx1_5	TRUE	—	—	1c	TRUE	
ERR127035	2a	TRUE	TRUE	gtrII, wzx1_5	TRUE	—	—	2a	TRUE	
ERR127036	2b	TRUE	TRUE	gtrII, gtrX, wzx1_5	TRUE	—	—	2b	TRUE	
ERR127037	3a	TRUE	TRUE	gtrX, oac, wzx1_5	TRUE	—	—	3a	TRUE	
ERR127038	3b	TRUE	TRUE	oac, wzx1_5	TRUE	—	—	3b	TRUE	
ERR127039	3b	TRUE	TRUE	oac, wzx1_5	TRUE	—	—	3b	TRUE	
ERR127040	4a	TRUE	TRUE	gtrIV, wzx1_5	TRUE	—	—	4a	TRUE	
ERR127041	4b	TRUE	TRUE	gtrIV, oac, wzx1_5	TRUE	—	—	4b	TRUE	
ERR127043	5a	TRUE	TRUE	gtrV, oac, wzx1_5	TRUE	—	oac	5a	TRUE	
ERR127046	X	TRUE	TRUE	gtrX, wzx1_5	TRUE	—	—	X	TRUE	
ERR127047	Y	TRUE	TRUE	wzx1_5	TRUE	—	—	Y	TRUE	
ERR200344	1b	TRUE	TRUE	gtrI, oac, wzx1_5	TRUE	——	—	1b	TRUE	
ERR200360	2a	TRUE	TRUE	gtrII, wzx1_5	TRUE	—	—	2a	TRUE	
ERR200365	2a	TRUE	TRUE	gtrII, wzx1_5	TRUE	—	—	2a	TRUE	
ERR200370	2a	TRUE	TRUE	gtrII, wzx1_5	TRUE	—	—	2a	TRUE	
ERR200378	2a	TRUE	TRUE	gtrII, wzx1_5	TRUE	—	—	2a	TRUE	
ERR200390	2b	TRUE	TRUE	gtrII, gtrX, wzx1_5	TRUE	—	—	2b	TRUE	
ERR200392	2b	TRUE	TRUE	gtrII, gtrX, wzx1_5	TRUE	—	—	2b	TRUE	
ERR200393	3a	TRUE	TRUE	gtrX, oac, wzx1_5	TRUE	—	—	3a	TRUE	
ERR200402	3a	TRUE	TRUE	gtrX, oac, wzx1_5	TRUE	—	—	3a	TRUE	
ERR200403	3a	TRUE	TRUE	gtrV, gtrX, oac, wzx1_5	TRUE	gtrV	gtrV	3a	TRUE	
ERR200405	3a	TRUE	TRUE	gtrV, gtrX, oac, wzx1_5	TRUE	gtrV	gtrV	3a	TRUE	
ERR200413	5a	TRUE	TRUE	gtrV, oac, wzx1_5	TRUE	—	oac	5a	TRUE	
ERR200414	Yv	TRUE	TRUE	gtrX, opt, wzx1_5	TRUE	—	gtrX	Yv	TRUE	
ERR217015	Yv	TRUE	TRUE	gtrX, opt, wzx1_5	TRUE	—	gtrX	Yv	TRUE	
ERR217016	Y	TRUE	TRUE	gtrX, wzx1_5	TRUE	—	gtrX	Y	TRUE	
ERR217023	Yv	TRUE	TRUE	gtrX, opt, wzx1_5	TRUE	—	gtrX	Yv	TRUE	
ERR217024	Y	TRUE	TRUE	gtrX, wzx1_5	TRUE	—	gtrX	Y	TRUE	
ERR217026	Y	TRUE	TRUE	gtrX, wzx1_5	TRUE	—	gtrX	Y	TRUE	
ERR217028	Yv	TRUE	TRUE	gtrX, opt, wzx1_5	TRUE	—	gtrX	Yv	TRUE	
ERR217030	Yv	TRUE	TRUE	gtrX, opt, wzx1_5	TRUE	—	gtrX	Yv	TRUE	
ERR217031	Y	TRUE	TRUE	gtrX, wzx1_5	TRUE	—	gtrX	Y	TRUE	
ERR217032	Yv	TRUE	TRUE	gtrX, opt, wzx1_5	TRUE	—	gtrX	Yv	TRUE	
ERR217047	1c	TRUE	TRUE	gtrI, gtrIC, wzx1_5	TRUE	—	—	1c	TRUE	
ERR217080	1c	TRUE	TRUE	gtrI, gtrIC, wzx1_5	TRUE	—	—	1c	TRUE	
ERR217081	4av	TRUE	TRUE	gtrIV, opt, wzx1_5	TRUE	—	—	4av	TRUE	
ERR217084	4av	TRUE	TRUE	gtrIV, opt, wzx1_5	TRUE	—	—	4av	TRUE	
ERR559526	2a	TRUE	TRUE	gtrII, wzx1_5	TRUE	—	—	2a	TRUE	
ERR832453	5a	TRUE	TRUE	gtrV, wzx1_5	TRUE	—	—	5a	TRUE	
ERR832456	2a	TRUE	TRUE	gtrII, wzx1_5	TRUE	—	—	2a	TRUE	
ERR832457	2a	TRUE	TRUE	gtrII, wzx1_5	TRUE	—	—	2a	TRUE	
ERR832459	5a	TRUE	TRUE	gtrV, oac, wzx1_5	TRUE	—	oac	5a	TRUE	
ERR832461	2b	TRUE	TRUE	gtrII, gtrX, wzx1_5	TRUE	—	—	2b	TRUE	
ERR832462	2a	TRUE	TRUE	gtrII, wzx1_5	TRUE	—	—	2a	TRUE	
ERR832464	5a	TRUE	TRUE	gtrV, oac, wzx1_5	TRUE	—	oac	5a	TRUE	
ERR832465	3b	TRUE	TRUE	oac, wzx1_5	TRUE	—	—	3b	TRUE	
ERR832467	2a	TRUE	TRUE	gtrII, wzx1_5	TRUE	—	—	2a	TRUE	
ERR832468	2a	TRUE	TRUE	gtrII, wzx1_5	TRUE	—	—	2a	TRUE	
ERR832470	1b	TRUE	TRUE	gtrI, oac, wzx1_5	TRUE	—	—	1b	TRUE	
ERR832474	Xv	TRUE	TRUE	gtrX, opt, wzx1_5	TRUE	—	—	Xv	TRUE	
ERR832477	3a	TRUE	TRUE	gtrX, oac, wzx1_5	TRUE	—	—	3a	TRUE	
ERR832483	1a	TRUE	TRUE	gtrI, wzx1_5	TRUE	—	—	1a	TRUE	
ERR832485	Y	TRUE	TRUE	gtrII, wzx1_5	TRUE	—	gtrII	Y	TRUE	
ERR832486	2b	TRUE	TRUE	gtrII, gtrX, wzx1_5	TRUE	—	—	2b	TRUE	
ERR832487	2a	TRUE	TRUE	gtrII, wzx1_5	TRUE	—	—	2a	TRUE	
ERR832489	2a	TRUE	TRUE	gtrII, wzx1_5	TRUE	—	—	2a	TRUE	
ERR832490	4bv	TRUE	TRUE	gtrIV, oac, opt, wzx1_5	TRUE	—	—	4bv	TRUE	
ERR832491	1b	TRUE	TRUE	gtrI, oac, wzx1_5	TRUE	—	—	1b	TRUE	
ERR832492	3b	TRUE	TRUE	oac, wzx1_5	TRUE	—	—	3b	TRUE	
ERR832494	X	TRUE	TRUE	gtrX, wzx1_5	TRUE	—	—	X	TRUE	
S14BD02502	X	TRUE	TRUE	gtrX, oac, wzx1_5	TRUE	—	—	3a	FALSE	oac indel not detected
S15BD08204	3b	TRUE	TRUE	gtrX, oac, wzx1_5	TRUE	—	—	3a	FALSE	promoter mutations not found
S13BD04017	3b	TRUE	TRUE	gtrX, oac, wzx1_5	TRUE	—	—	3a	FALSE	promoter mutations not found
S14BD01131	X	TRUE	TRUE	gtrX, oac, wzx1_5	TRUE	—	oac	X	TRUE	
S14BD01142	X	TRUE	TRUE	gtrX, oac, wzx1_5	TRUE	—	oac	X	TRUE	
S14BD01714	3b	TRUE	TRUE	gtrX, oac, wzx1_5	FALSE	—	—	3b	TRUE	
S15BD06353	3b	TRUE	TRUE	gtrX, oac, wzx1_5	TRUE	—	—	3a	FALSE	promoter mutations not found
S15BD09453	3b	TRUE	TRUE	gtrI, oac, wzx1_5	TRUE	—	—	1b	FALSE	Indel in gtrI at position 340 not detected
S16BD02240	5	FALSE	TRUE	gtrV, wzx1_5	TRUE	—	—		TRUE	
S17BD07654	x	TRUE	TRUE	gtrX, oac, oac, wzx1_5	TRUE	—	—	3a	FALSE	oac indel not detected

^a^As determined by classical serotyping methods.

^b^Based on comparison with the gtr promoter (accession number KT988057.1).
